# The Osmoprotectant Switch of Potassium to Compatible Solutes in an Extremely Halophilic Archaea *Halorubrum kocurii* 2020YC7

**DOI:** 10.3390/genes13060939

**Published:** 2022-05-24

**Authors:** Runting Ding, Na Yang, Jianguo Liu

**Affiliations:** 1 CAS and Shandong Key Laboratory of Experimental Marine Biology, Center for Ocean Mega-Science, Institute of Oceanology, Chinese Academy of Sciences, 7 Nanhai Road, Qingdao 266071, China; dingrunting@qdio.ac.cn (R.D.); yangna@qdio.ac.cn (N.Y.); 2 University of Chinese Academy of Sciences, Beijing 100049, China; 3 Laboratory for Marine Biology and Biotechnology, Qingdao National Laboratory for Marine Science and Technology, 1 Wenhai Road, Qingdao 266237, China

**Keywords:** halophilic archaea, *Halorubrum kocurii* 2020YC7, osmoprotectant strategy, potassium, glycine betaine, trehalose

## Abstract

The main osmoadaptive mechanisms of extremely halophilic archaea include the “salt-in” strategy and the “compatible solutes” strategy. Here we report the osmoadaptive mechanism of an extremely halophilic archaea *H. kocurii* 2020YC7, isolated from a high salt environment sample. Genomic data revealed that strain 2020YC7 harbors genes *trkA*, *trkH*, *kch* for K^+^ uptake, *kefB* for K^+^ output, *treS* for trehalose production from polysaccharide, and betaine/carnitine/choline transporter family gene for glycine betaine uptake. Strain 2020YC7 could accumulate 8.17 to 28.67 μmol/mg protein K^+^ in a defined medium, with its content increasing along with the increasing salinity from 100 to 200 g/L. When exogenous glycine betaine was added, glycine betaine functioned as the primary osmotic solute between 200 and 250 g/L NaCl, which was accumulated up to 15.27 mg/mg protein in 2020YC7 cells. RT-qPCR results completely confirmed these results. Notably, the concentrations of intracellular trehalose decreased from 5.26 to 2.61 mg/mg protein as the NaCl increased from 50 to 250 g/L. In combination with this result, the transcript level of gene *treS*, which catalyzes the production of trehalose from polysaccharide, was significantly up-regulated at 50–100 g/L NaCl. Therefore, trehalose does not act as an osmotic solute at high NaCl concentrations (more than 100 g/L) but at relatively low NaCl concentrations (50–100 g/L). And we propose that the degradation of cell wall polysaccharide, as a source of trehalose in a low-salt environment, may be one of the reasons for the obligate halophilic characteristics of strain 2020YC7.

## 1. Introduction

Halophilic archaea are microorganisms that can live in a high-salt environment, such as salt lakes, salt farms, pickled food, and other environments [1]. Due to the NaCl concentration being 2.5–5.2 M, most halophilic archaea are classified as extremely halophilic microorganisms [2]. To live in hypersaline environments, osmoadaptive strategies are employed by extremely halophilic microorganisms through increasing the osmotic activity of cytoplasm, which is of general biological interest.

It has been reported that halophilic archaea can survive in a high-salt environment due to the high proportion of acidic amino acids, the synthesis of bacterioruberin (a C50 carotenoid) and two osmoadaptive strategies. A high proportion of acidic amino acids, causes the intracellular proteins to bind more K^+^ [3,4,5]. Bacterioruberin could increase the rigidity of cell membranes by reducing fluidity as well as exert an anti-stress effect and was regulated by salinity [6,7,8]. The main osmoadaptive mechanisms included the “salt-in” strategy and the “compatible solutes” strategy [2,5,9,10,11]. The “salt-in” strategy is based on the input of K^+^, which balanced the high concentration of Na^+^ in the high-salt medium [12,13]. Series of enzymes and transport systems are activated and/or induced by K^+^, which acts as a signal molecule to adapt to the changing osmotic pressure [14]. Common compatible solutes in extremely halophilic archaea include amino acids and their derivatives, polyols, carbohydrates, etc. [15,16]. Studies had shown that organic solutes trehalose and glycine betaine were widespread in the extremely halophilic archaea by *de novo* synthesis or uptake from environment [17,18]. Trehalose is an important component that has both osmoadaptive regulation and cell protection effects in halophilic archaea [19]. There are 5 pathways for the biosynthesis of trehalose, including OstAB, TreS, TreP, TreY and TreT pathway, respectively [20]. The OstAB pathway is ubiquitous in halophilic archaea, such as *Halococcus saccharolyticus*, *Halogranum salarium*, *Natrinema pellirubrum*, etc. And halophilic archaeal species with *ostAB* gene have lower optimal growth salinity than those lacking this pathway [19]. Glycine betaine is another widely used solute by many species, including most archaea, bacteria, plants and humans, etc [21,22]. The *de novo* synthesis pathway of glycine betaine generally used choline or glycine as a precursor [23,24].

K^+^ is the first choice for osmoadaptive regulation due to the ubiquitous transport system, high transport efficiency and good regulation effect [25]. The increase of K^+^ is an early response to a high-salt environment in eubacteria [26,27]. The main osmoprotectants change from K^+^ to organic compatible solutes over time, such as proline and glycine betaine [28]. Therefore, eubacteria tend to use zwitter ionic or nonionic solutes to balance osmotic pressure with the outside world. During the transition from moderate salinity (around 1 M NaCl) to high salinity (2 to 3 M NaCl), the main osmoprotectant of moderately halophilic bacterium *Halobacillus halophilus* was converted from glutamate to proline [29]. Furthermore, the main osmoprotectant switch from K^+^ to glycine betaine has also been found in extremely halophilic bacteria *Halorhodospira halophila* when the K^+^ concentration in the environment was less than 1 g/L [30]. Conversely, in archaea, the K^+^ concentration is relatively stable in a high-salt environment, which makes K^+^, rather than compatible solutes, considered as the main osmoprotectant [2,15]. In recent years, it has been found that genes for trehalose synthesis and glycine betaine transport are widespread in halophilic archaea, and the intracellular K^+^ concentration decreased after the addition of glycine betaine [17]. This raises the question of whether a complex switching mechanism of osmoprotectants also exists in halophilic archaea.

The current researches of halophilic mechanism of halophilic archaea mainly focuses on the physiological level detection of intracellular osmoaprotectants [17,18,31,32,33]. Few gene-level and global issues were addressed and few research advances were put into practice. In this study, the osmoadaptive mechanism of an extremely halophilic archaea *H. kocurii* 2020YC7 was studied. The whole genome of *H. kocurii* 2020YC7 has been sequenced, and genes related to the osmoadaptive regulation have been mined. After the addition of trehalose and glycine betaine in different salinities, the physiological levels of trehalose and glycine betaine have been measured, and the expression levels of related genes have been analyzed. This study aims to perform a more systematic and in-depth exploration of the osmoadaptive mechanism and discuss the mutual influence of each part of the osmoadaptive mechanism.

## 2. Materials and Methods

### 2.1. Archaeal Strain and Growth Condition

*H. kocurii* 2020YC7 was isolated from high salt water (Northern China) and grown in YCM1 medium (MgSO_4_·7H_2_O 20 g/L, KCl 2 g/L, sodium citrate tribasic dihydrate 3 g/L, oxoid yeast extract 3 g/L, tryptone 5 g/L, pH = 7.0). For osmoadaptive experiments, media YCM2 (Tris-Cl 50 mM, glucose 10 g/L, pH = 7.0) was used. The NaCl concentrations of both YCM1 and YCM2 were 50, 100, 150, 200, and 250 g/L.

### 2.2. Genomic Sequencing

The genome of *H. kocurii* strain 2020YC7 was sequenced using PacBio Sequel platform and Illumina NovaSeq PE150 (Novogene, Beijing, China). Genomic DNA was extracted with the SDS method (Lim, Lee, Yoon, Chua, & Son, 2016). The harvested DNA was detected by the agarose gel electrophoresis and quantified by Qubit 2.0 Fluorometer (Thermo Fisher Scientific, Waltham, MA, USA). Preliminary assembly Program SMRT Link v5.0.1 was used to preliminary gene assembly. By the variant Caller module of the SMRT Link software, the arrow algorithm was used to correct and count the variant sites in the preliminary assembly results. The corrected assembly result, which was used as the reference sequence, was blasted with Illumina data by bwa. Futhermore, the result was filtered with the base minimum mass value of was 20, the minimum read depth of 4 and the maximum read depth of 1000. Based on the overlap between the head and the tail, we examined whether the chromosomal sequence formed a circle or not, then corrected the initial site by blasting with the DNA database. Finally, the chromosome and plasmid sequences were screened by blast with plasmid database. Six databases were used to predict gene functions. They were GO (Gene Ontology) [34], KEGG (Kyoto Encyclopedia of Genes and Genomes) [35], COG (Clusters of Orthologous Groups) [36], Non-Redundant Protein Database (NR) [37], TCDB (Transporter Classification Database) [38], and Swiss-Prot [39]. A whole genome Blast search (E-value less than 1 × 10^−5^, minimal alignment length percentage larger than 40%) was performed against above seven databases.

### 2.3. Phylogenetic Analysis

Homologous gene sequence search was completed by BLAST algorithm at the National Center for Biotechnology Information (http://www.ncbi.nlm.gov/blast/) (accessed on 23 February 2022) [40]. Gene multiple sequence alignment was done by DNAMAN and ClustalW Multiple Alignment program (http://www.ebi.ac.uk/clustalw/) (accessed on 23 February 2022) [41]. The phylogenetic tree was constructed using the maximum likelihood (ML) algorithm method by the MEGA 7 program [42]. The reliability of the branching was tested by 1000 bootstraps.

### 2.4. Reverse Transcription qPCR (RT-qPCR) Analysis

The primers’ information of eight osmoadaptive -related genes was shown in Table S1, and primers were synthesized by a company (Sangon, Shanghai, China). The 16s rRNA gene of *H. kocurii* 2020YC7 was chosen as reference gene for internal standard-dization. Each gene was normalized using the CT value of the 50 g/L group in the same group of treatments. Total RNA extraction was completed from strains cultured for 24 h with a bacterial total RNA extraction kit (Insight, Qingdao, China). Total RNA reverse transcription was performed according to the protocol using a ReverTra Ace qPCR RT Msater Mix (TOYOBO, Osaka, Japan). The total RNA extracted and cDNA reverse transcribed were confirmed by agarose gel electrophoresis. Reverse transcription qPCR (RT-qPCR) reaction system consisted of cDNA templates, primers, and TOROGreen 5G qPCR premix (Toroivd, Shanghai, China). The RT-qPCR was carried out in a total volume of 20 μL, containing 10 μL TOROGreen qPCR premix, 2 μL cDNA template, 4 μL forward primer and 4 μL reverse primer. The process adopted a two-step method. Thermal cycling conditions were an initial denaturation step at 92 °C for 2 min followed by 40 cycles consisting of denaturation at 95 °C for 3 s and primer annealing and strand extension at 60 °C for 20 s. RT-qPCR was completed by QuantStudio 6 Flex (Thermo Fisher, Waltham, MA, USA), and sequencing results collection and analysis were also completed by this equipment. The results are analyzed using the ΔΔCt method.

### 2.5. Determination of Biomass and Bacterioruberin Accumulation

Biomass and bacterioruberin content were both measured by UV spectrophotometry. Biomass was measured at 600 nm absorbance in the media YCM1 with 50, 100, 150, 200, 250 g/L NaCl per 24 h. Bacterioruberin was extracted by methanol and then determined at 494 nm absorbance. The ratio of A_494_ to A_600_ was used to express the bacteriocin content of unit cells.

### 2.6. Determination of Intracellular K^+^, Trehalose and Glycine Betaine Concentration

The archaea cultured to the stationary phase (A_600_ value no longer changed with time, about 0.5) in YCM1 media was collected and transferred to YCM2 medium after being washed 3 times with 250 g/L NaCl solution. Cells were cultivated in different salinities in the media YCM2 for 24 h with or without adding extra 1% trehalose and 1% glycine betaine. Cells were lysed by ddH_2_O. Osmoprotectants were identified and quantified in cell-free extracts. Concentration of intracellular K^+^, trehalose and glycine betaine measured using K^+^ turbid assay kit, colorimetric trehalose assay kit and glycine betaine assay kit (Solarbio, Beijing, China). The principle of K^+^ detection was that K^+^ reacted with Na_2_B_4_O_7__·_xH_2_O to produce K_2_B_4_O_7_·5H_2_O which was insoluble in water, and the turbidity was proportional to the K^+^ concentration within a certain range. The detection of trehalose utilized the anthrone spectrophotometric methods. The detection principle of glycine betaine was that glycine betaine reacted with Reinecke salt under strong acid conditions to form a precipitate, which can be dissolved in acetone to form a red solution with a characteristic absorption peak at 525 nm.

### 2.7. Statistical Analysis

Each experiment was repeated three times. Statistical analysis was performed by SPSS 16.0 (Chicago, IL, USA). The variation of each treatment was tested by one-way analysis of variance (ANOVA) procedure. The differences between groups were analyzed by least significant difference (LSD). Differences were considered significant at a probability level of *p* < 0.05.

## 3. Results and Discussion

### 3.1. General Features

**Figure 1:**
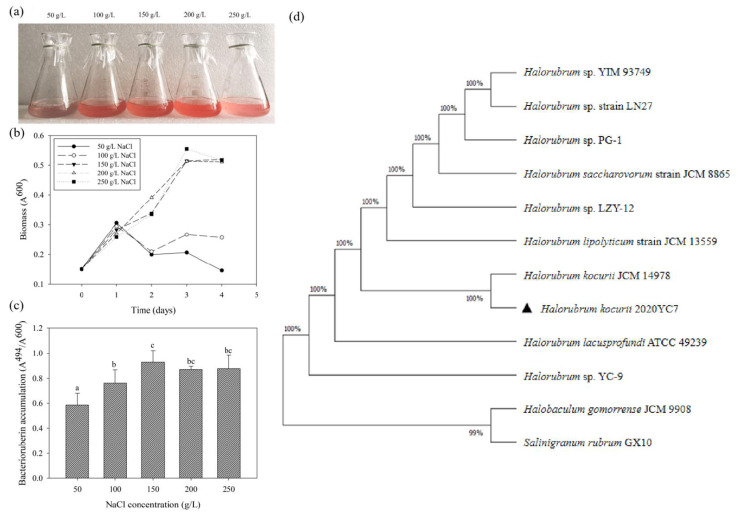
Growth status after 48 h incubation (**a**) in YCM2 medium, growth curve (**b**) and bacterioruberin accumulation (**c**) in YCM1 medium of *H. kocurii* 2020YC7 at 50, 100, 150, 200, 250 g/L NaCl concentration. Maximum likelihood (ML) algorithm method phylogenetic tree (**d**), based on 16s rRNA gene sequence of *H.kocurii* 2020YC7 (“▲” marked), and compared with *Halorubrum* sp. YIM 93749 (JF449434) *Halorubrum* sp. strain LN27 (MN829451), *Halorubrum* sp. PG-1 (KJ644278), *Halorubrum saccharovorum* strain JCM 8865 (NR_113484), *Halorubrum* sp. LZY-12 (KJ644292), *Halorubrum lipolyticum* strain JCM 13559 (NR_113480), *H. kocurii* JCM 14978 (NR_113215), *Halorubrum lacusprofundi* ATCC 49239 (NR_074194), *Halorubrum* sp. YC-9 (JN216854), *Halobaculum gomorrense* JCM 9908 (AB477983), *Salinigranum rubrum* GX10 (GU951431).

Growth media with different concentrations of NaCl (50, 100, 150, 200, 250 g/L) were used for the cultivation of strain 2020YC7. The optimal growth condition for strain 2020YC7 was 150–250 g/L (Figure 1a,b). Based on this result, strain 2020YC7 is an extremely halophilic archaea [16]. The bacterioruberin content increased with increasing NaCl concentrations. Compared with the 50 g/L group, the accumulation of bacteriocin in the 250 g/L group was 1.58 times higher. (Figure 1a,c). Maximum likelihood (ML) algorithm method was performed to build the phylogenetic tree of strain 2020YC7 (Figure 1d). Strain 2020YC7 was compared with some species of the same genus and another two strains belonging to the same family based on the 16S rRNA sequences. Strain 2020YC7 had the highest similarity to the *H. kocurii* JCM 14978 sequences with a similarity value of 100%.

### 3.2. Genome Features and Osmoadaptive Genes

The total length of *H. kocurii* 2020YC7 genome was 3,727,755 bp. The information of the chromosome is shown in Table 1. The GC content of the chromosome was up to 68.22%, which made its DNA more stable in a hypertonic environment. The GC content of halophilic archaea genomes was generally high, such as *H. kocurii* BG-1 (69.4%), *H. saccharovorum* NCIMB 2081 (71.2%) and *H. lacusprofundi* ACAM 34 (65.3%) [43,44]. In contrast, the NCBI data (http://www.ncbi.nlm.nih.gov/) showed that the GC content of non-halophilic *Escherichia coli* was 50.4%. *H. kocurii* 2020YC7 contains a linear chromosome and 3 linear plasmids. By gathering the genome information of various halophilic and non-halophilic species, the proportion of acidic amino acids in *H. kocurii* 2020YC7 was higher (17.14%) than that of non-halophilic and halophilic species (*Halobacterium salinarum* NRC-1 13.20%, *Haladaptatus paucihalophilus* DX253 12.87%, *Halobacterium hubeiense* 13.97%, *Nesterenkonia alba* DSM 19423 12.97%, *Salinicoccus albus* DSM 19776 11.50%, *Staphylococcus agnetis* 10.23%, *Dunaliella salina* 9.15% and *E. coli* str. K-12 substr. MG1655 8.93%). The higher proportion of acidic amino acids indicated that more cations for osmotic regulation could be combined in strain 2020YC7 cells [3].

Based on the genome results, a total of 34 genes related to osmoadaptive regulation were identified in strain 2020YC7 (Table 2). The genes included Trk system (*trkA* and *trkH*) and K^+^ voltage-gate channel (*kch*) for K^+^ uptake, K^+^/H^+^ antiporter (*kefB*) for potassium discharge. Genes for the synthesis or uptake of trehalose and glycine betaine were also detected in the genome of *H. kocurii* 2020YC7, including amylase/trehalose synthase (*treS*), sugar ABC transporter (*sugA*), and betaine/carnitine/choline transporter (*bcct*). The genes used for analysis in the article had been deposited in the NCBI database with GenBank numbers (Table 2). Becker et al. performed a phylogenetic analysis of the osmoadaptative strategies of extreme halophilic archaea and constructed a generalized model. The results showed that the Trk uptake system, the Kef anti-transporter and the NhaC anti-transporter played a pervasive function in the osmoregulation strategy of halophilic archaea [45].

**Figure 2:**
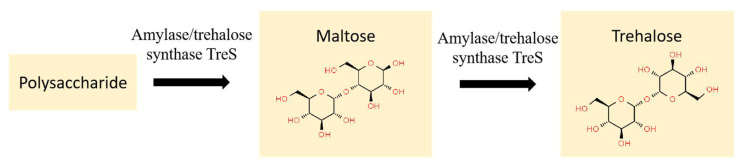
The trehalose synthesis pathway of *treS* in *H. kocurii* 2020YC7.

The Trk system of *H. kocurii* 2020YC7 included TrkA and TrkH without TrkE [46,47]. Studies have shown that although TrkE binds ATP, ATP may only play a regulatory role and not provide energy for the transport process [48]. In many species, the presence of TrkE protein was not necessary and may be replaced by other ABC transporters [31,49]. TreS can catalyze polysaccharide to maltose and then transform it to trehalose (Figure 2) [50,51]. Strain *H. kocurii* 2020YC7 could use other types of polysaccharides for the production of trehalose, as no starch was detected using the iodine method in our study, which needs further study [52].

### 3.3. Osmoadaptive Mechanisms without Exogenous Compatible Solutes

**Figure 3:**
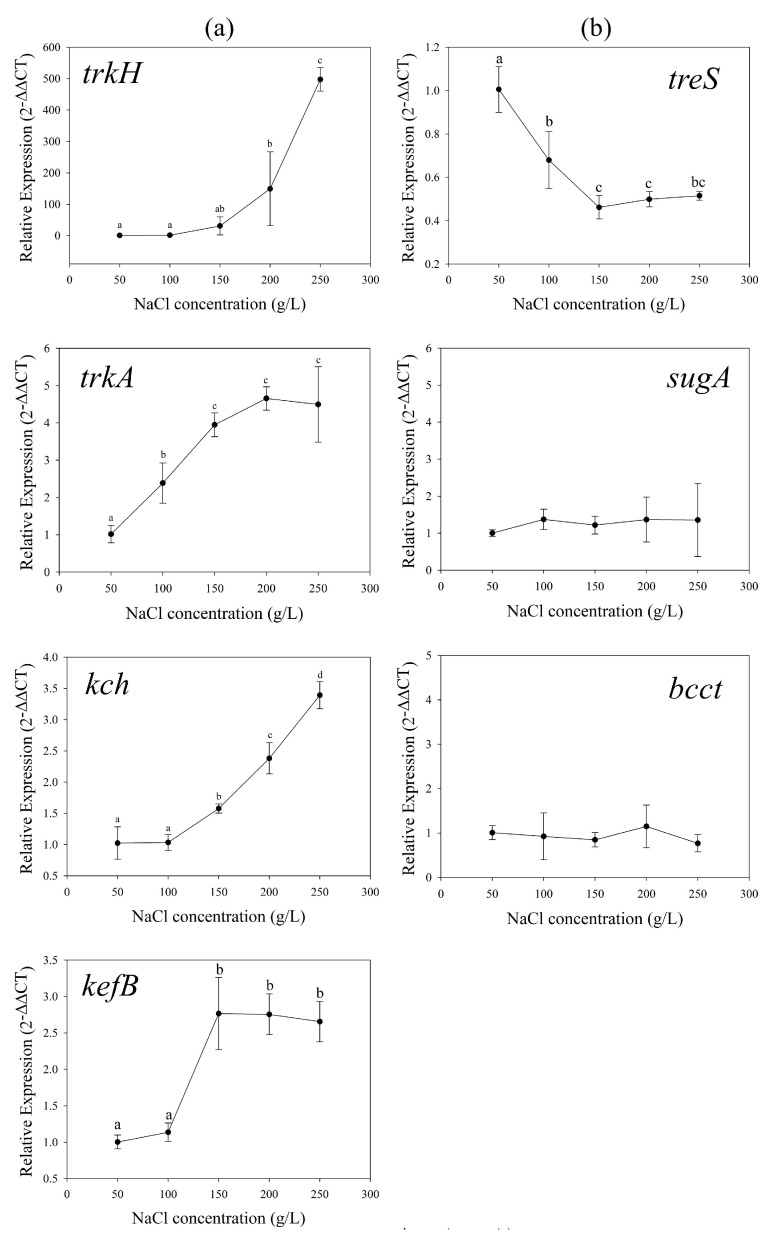
The relative expression levels of potassium uptake genes *trkH*, *trkA*, *kch* and output gene *kefB* (**a**), amylase/trehalose synthesis gene *treS*, compatible solutes transport genes *sugA* and BCCT family gene (**b**) in different salt concentration without exogenous compatible solutes.

The relative expression levels of K^+^ uptake genes *trkH*, *trkA*, *kch*, and *kefB* were detected. Results showed that the transcripts of all those genes increased with increasing NaCl concentrations (Figure 3a). Compared with *kch*, gene expression of Trk system was more strongly affected by salinity. Especially *trkH*, the relative expression level in 250 g/L group was 500 times higher than 50 g/L group. It showed the Trk potassium uptake system played a significant role during the osmoadaptive regulation in *H. kocurii* 2020YC7, which is consistent with other species of halophilic archaea [14,47,53]. The expression level of amylase/trehalose synthesis gene *treS* was also examined and decreased with the increasing NaCl concentrations (Figure 3b). The relative expressions in 50, 100, 150, 200, and 250 g/L NaCl concentration were 1.00, 0.68, 0.46, 0.50, and 0.51, respectively. Without external supplementation, the transcripts of glycine betaine transport gene *bcct* and trehalose transport gene *sugA* did not change (Figure 3b).

**Figure 4:**
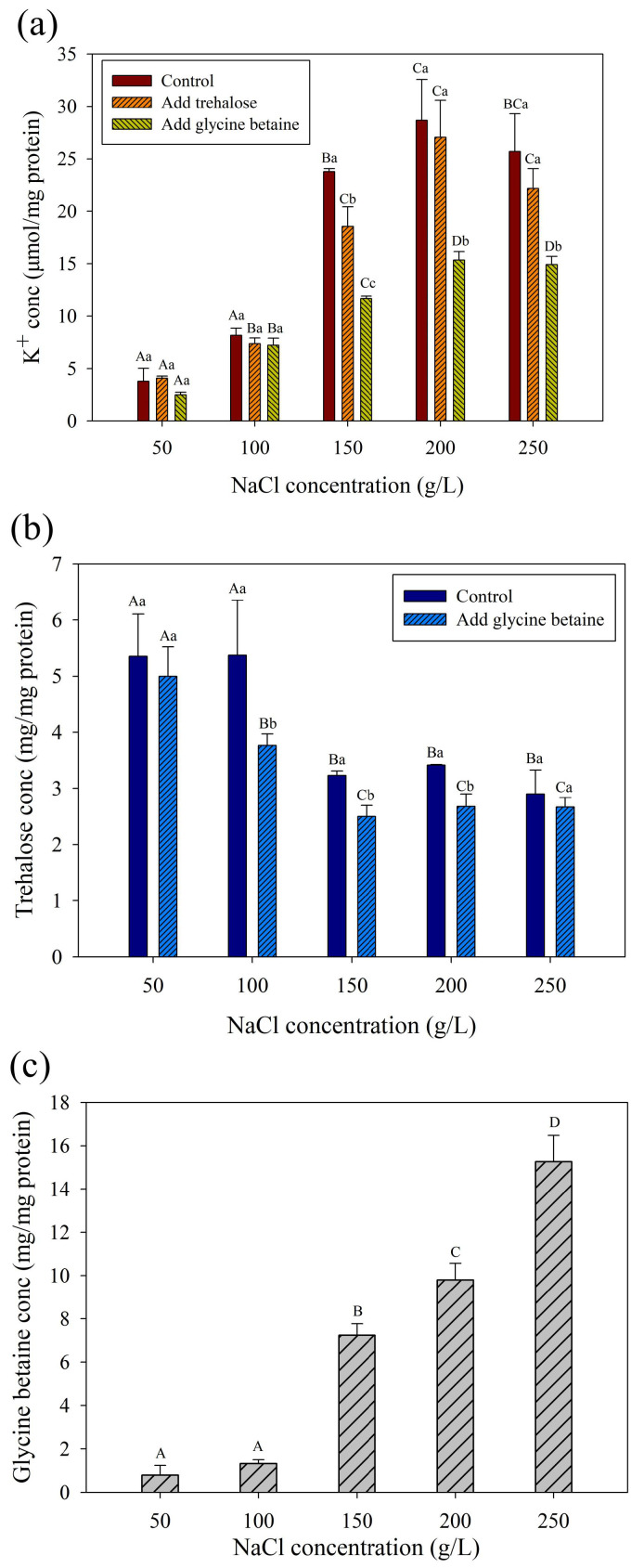
Intracellular K^+^ (**a**), trehalose (**b**) and glycine betaine (**c**) concentration changes with the different NaCl concentrations. “A, B, C, D” refers to the significance analysis between groups with different NaCl concentrations (50–250 g/L). “a, b, c, d” refers to the significance analysis between groups with no extras, add 1% extra trehalose and add 1% glycine betaine.

The uptake of K^+^ was considered to be the most important osmoadaptive way for halophilic archaea [17,54,55,56,57,58,59]. As is shown in Figure 4a, the intracellular K^+^ concentration increased with increasing NaCl concentrations. The intracellular K^+^ concentration increased significantly in cells with 150–250 g/L NaCl compared to those in cells with 50–100 g/L (*p* < 0.05). The K^+^ concentration in 200 g/L group reached up to 28.67 μmol/mg protein which was 7.5 times higher than that in 50 g/L group, indicating that K^+^ uptake was an important way for *H. kocurii* 2020YC7 to regulate osmotic pressure. Since the genome showed that *H. kocurii* 2020YC7 had the amylase/trehalose synthesis gene *treS*, we detected the trehalose content in its cells. Trehalose concentration decreased with increasing NaCl concentrations (Figure 4b) which was consistent with the relative expression change trend of *treS*. Trehalose concentration decreased from 5.00 to 2.67 mg/mg protein during the NaCl concentration increasing from 50 to 250 g/L. No gene related to glycine betaine synthesis was found in the genome, and no endogenous glycine betaine was detected in the cells. It meant that glycine betaine cannot be *de novo* synthesized by *H. kocurii* 2020YC7.

### 3.4. Osmoadaptive Mechanisms with Addling Exogenous Compatible Solutes

Glycine betaine was one of the most widespread compatible solutes, and was originally discovered to be utilized by halophilic eubacteria [60,61,62]. Its transporters (BCCT family of secondary transporters and QAT family of ABC transporters) were later found to be widespread in halophilic archaea taxa and validated in *H. salinarum* NRC-1 [17]. We found two copies of BCCT family transporters (OM942798 and OM942799) in the genome of *H. kocurii* 2020YC7. At the same time, halophilic cyanobacteria also exist in the high salt environment where strain 2020YC7 lived. It can synthesize glycine betaine as an osmoprotectant and release it into the environment [12]. Therefore, we proposed that strain 2020YC7 could uptake glycine betaine released by other organisms co-lived in the high salt environment.

**Figure 5:**
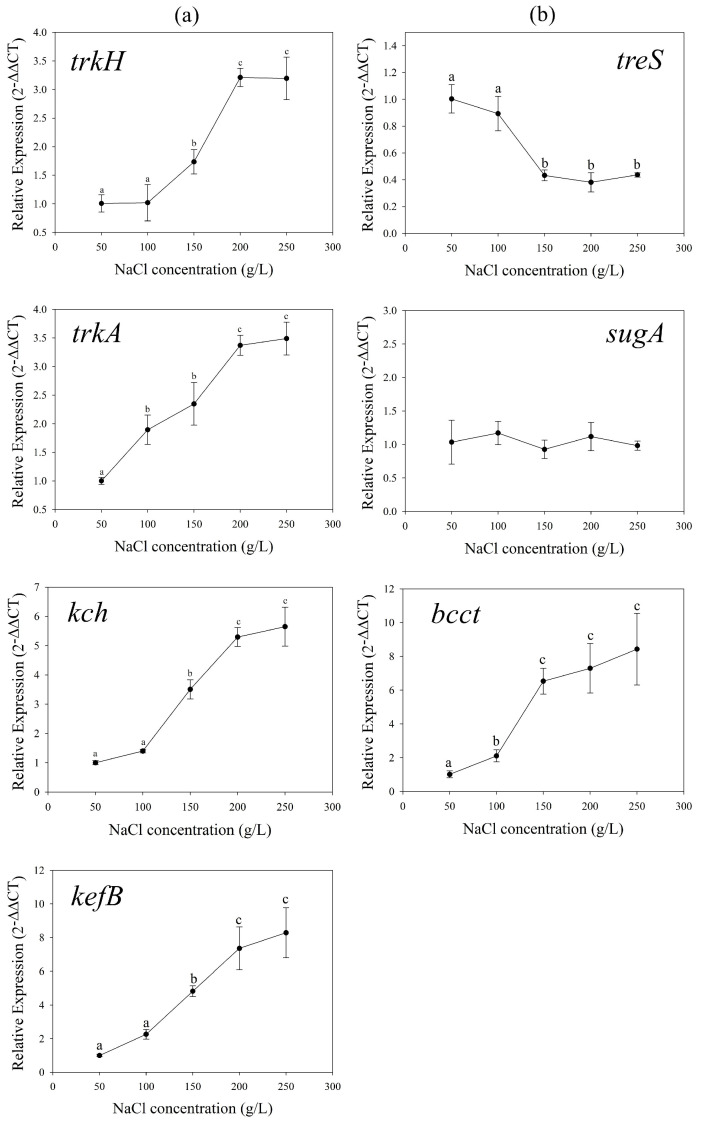
The relative expression levels of potassium uptake genes *trkH*, *trkA*, *kch* and output gene *kefB* (**a**), amylase/trehalose synthesis gene *treS*, compatible solutes transport genes *sugA* and BCCT family gene (**b**) in different salt concentration with adding exogenous glycine betaine.

When exogenous glycine betaine was supplied, the relative expression levels of potassium transport genes *trkH*, *trkA*, *kch* and *kefB* still increased with the increasing NaCl concentrations, but the amplitude of *trkH* decreased. (Figure 5a). We noted that with glycine betaine, there was no difference in the expression of *trkH* between 200 and 250 g/L, which was different from the treatment without exogenous addition (Figure 5a). The relative expression level of the amylase/trehalose synthesis gene *treS* also showed a downward trend. In these two BCCT family gene copies, only OM942799 was detected by RT-qPCR method. The relative expression of the glycine betaine transport gene *bcct* showed an obvious upward trend with increased salinity (Figure 5b).

After exogenous glycine betaine was added, both intracellular K^+^ and trehalose concentrations decreased significantly compared to the treatment without any exogenous compatible solutes in 150–250 g/L NaCl (*p* < 0.05) (Figure 4a,b). Intracellular glycine betaine was detected in the cells of *H. kocurii* 2020YC7. And the glycine betaine concentration increased with increasing NaCl concentrations, with a maximum of 15.27 mg/mg protein detected at 200 g/L (Figure 4c). Combined with expression analysis of potassium and glycine betaine transport genes, it was speculated that when the NaCl concentration was higher than 200 g/L, osmoadaptative functions of some K^+^ were replaced by exogenous glycine betaine. The changes of K^+^ concentration showed that the osmoadaptative mechanism of *H. kocurii* 2020YC7 was the combination of glycine betaine and K^+^, rather than relying entirely on a single substance when the K^+^ concentration was 2 g/L. “Compatible solute” strategy was more flexible than “salt-in” strategy in terms of osmoregulation. Therefore, microorganisms that can utilize compatible solutes to cope with osmotic stress can adapt to a wider range of salinity [63]. This allowed the main osmoadaptative mechanism to switch from K^+^ uptake to glycine betaine transport in the presence of glycine betaine in strain 2020YC7. Due to the presence of glycine betaine in its natural growth environment, it was the result of evolution that *H. kocurii* 2020YC7 did not have the ability to synthesize glycine betaine like other halophilic archaea species but had evolved a powerful BCCT family transport system.

**Figure 6:**
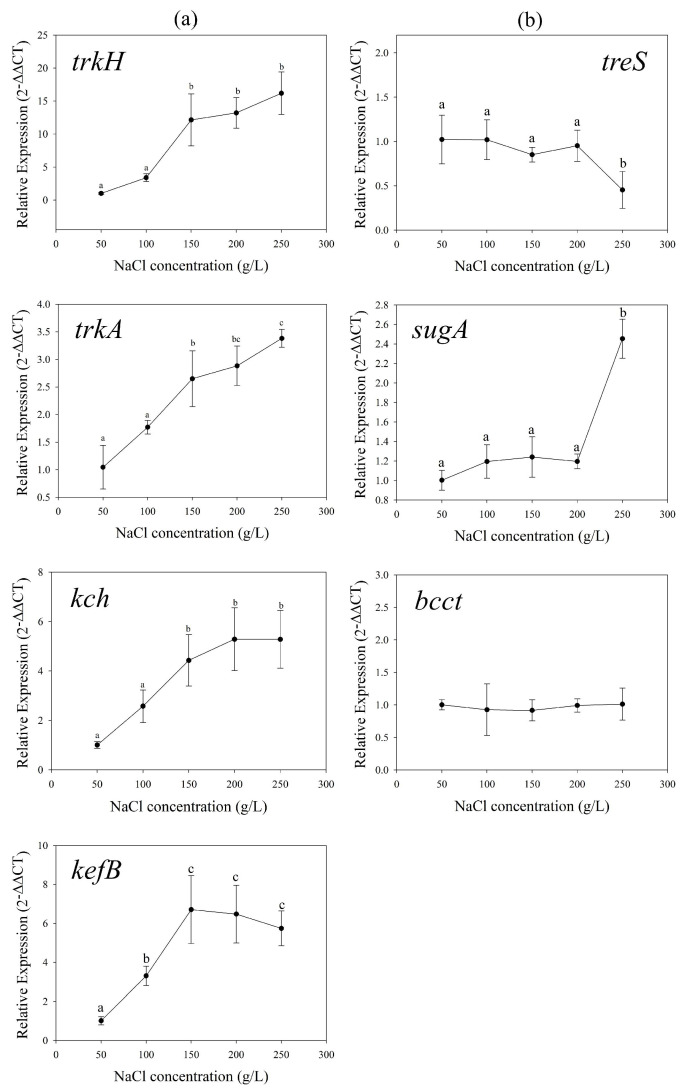
The relative expression levels of potassium uptake genes *trkH*, *trkA*, *kch* and output gene *kefB* (**a**), amylase/trehalose synthesis gene *treS*, compatible solutes transport genes *sugA* and BCCT family gene (**b**) in different salt concentration with adding exogenous trehalose.

Due to the presence of *sugA* in the genome, we also added exogenous trehalose for experiments. When added trehalose, the relative expression of potassium transport genes *trkH*, *trkA*, *kch*, *kefB* still showed an upward trend. The relative expression of amylase/trehalose synthesis gene *treS* also decreased with the increase of NaCl concentration. The expression of the trehalose transport gene *sugA* was not significantly changed in 50–200 g/L salinity groups (*p* > 0.05), whereas had a 2.5-fold increase in 250 g/L NaCl concentration group compared to 50 g/L group (Figure 6b).

Unlike glycine betaine, the addition of trehalose did not significantly decrease the intracellular K^+^ concentration at 50, 100, 200, 250 g/L NaCl groups (*p* > 0.05) (Figure 6a,b). This indicated that *H. kocurii* 2020YC7 was not inclined to absorb trehalose for performing osmotic regulation in conjunction with K^+^. These results suggested that strain 2020YC7 preferred to transport glycine betaine rather than trehalose from the environment. The decreased trehalose concentration with increasing salinity has been validated in *H. paucihalophilus* [17] and *H. hamelinensis* [18]. These studies suggested that the reason for the decrease of trehalose content with NaCl concentration was that the energy cost of synthesizing trehalose was higher than other osmoadaptive mechanisms [12,17]. However, combined with the changing trend of K^+^ concentration after the addition of trehalose, trehalose did not act as an osmoadaptative solute at high NaCl concentrations (more than 100 g/L) but at relatively low NaCl concentrations (50–100 g/L). Trehalose was derived from polysaccharides in strain 2020YC7. In a low-salt environment, polysaccharide breakdown caused cells collapse, then trehalose was released. This explained why strain 2020YC7 cannot survive in a low-salt environment from another perspective.

**Figure 7:**
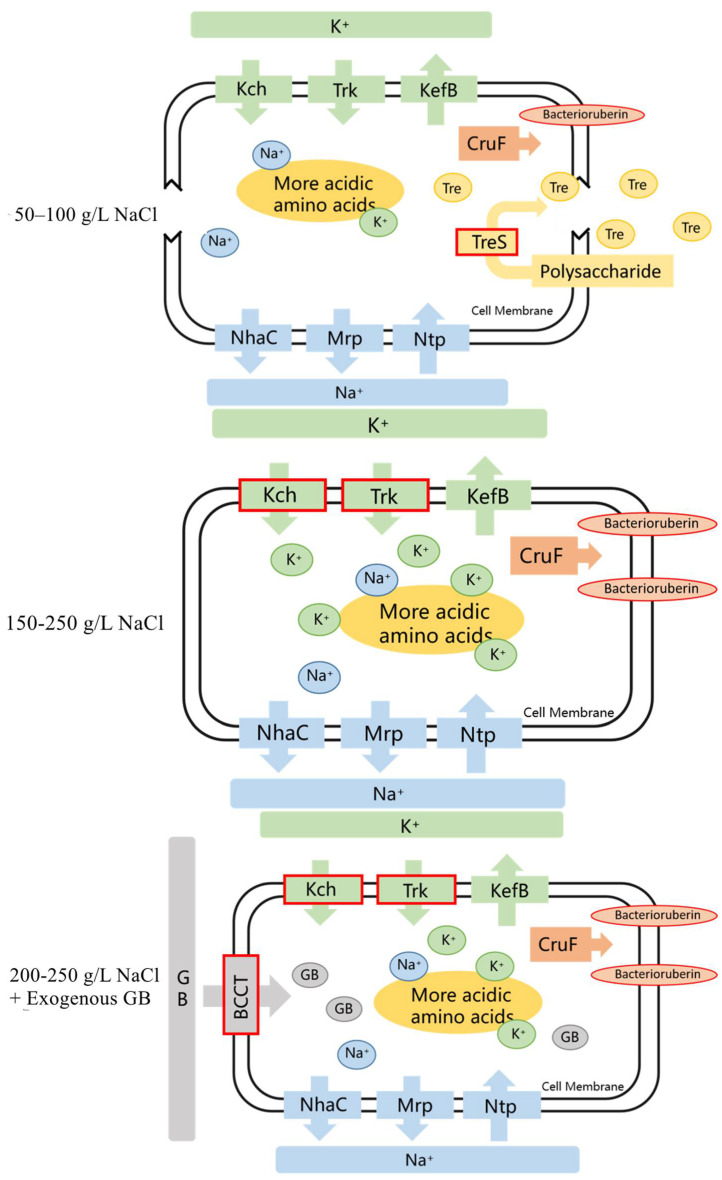
Overview of the halophilic mechanism of halophilic archaea *H. kocurii* 2020YC7. “GB” referred to glycine betaine and “Tre” referred to trehalsoe. “CruF” referred to carotenoid 1,2-hydratase.

In total, the osmoadaptive strategy of *H.* *kocurii* 2020YC7 was first “K^+^ - in”, second glycine betaine uptake, and finally, the de novo biosynthesis of trehalose via *treS*. The osmoadaptative mechanism of *H. kocurii* 2020YC7 was summarized as Figure 7.

## 4. Conclusions

Uptake of K^+^ and glycine betaine and biosynthesis of trehalose were the main osmoadaptive mechanisms in halophilic archaea *H. kocurii* 2020YC7. The transporters of K^+^ and glycine betaine were activated at an external NaCl concentration between 100–150 g/L. Uptake of K^+^ was the main osmoadaptative mechanism employed by 2020YC7 at 100–200 g/L NaCl. With exogenous addition of glycine betaine, glycine betaine functioned as the primary osmotic solute while intracellular K^+^ declined between 200 and 250 g/L NaCl. The intracellular content of trehalose was significantly increased at 50–100 g/L NaCl, indicating the enhanced degradation of polysaccharides in a relatively hypotonic environment.

## Figures and Tables

**Table 1:** General features of *Halorubrum* sp. 2020YC7 genome.

Feature	Chromosome Characteristics
Genome topology	circular
Genome size (bp)	3,085,069
G+C content (%)	68.22
Protein coding genes	3513
tRNA genes	48
rRNA operons	5 s, 16 s, 23 s

**Table 2:** Genes for uptake and discharge of potassium and sodium, biosynthesis and uptake of trehalose, and uptake of glycine betaine in *H. kocurii* 2020YC7.

Function	GenBank Number
**Potassium uptake**	
Trk potassium uptake system	OM942767, OM942768, OM942769, OM942770, OM942771, OM942772, OM942773, OM942774, OM942775, OM942776, OM942777, OM942778
Potassium voltage gate channel (*kch*)	OM942779
**Potassium discharge**	
Cation:proton antiporter (*kefB*)	OM942780
**Sodium uptake**	
V-type sodium ATPase (*ntp*)	OM942781, OM942782, OM942783, OM942784, OM942785
**Sodium discharge**	
Na^+^/H^+^ antiporter (*nhaC*)	OM942786
Multicomponent Na+:H+ antiporter (*mrp*)	OM942787, OM942788, OM942789, OM942790, OM942791, OM942792, OM942793, OM942794, OM942795
**Trehalose biosynthesis**	
Amylase/trehalose synthase (*treS*)	OM942796
**Trehalose uptake**	
Sugar ABC transporter permease (*sugA*)	OM942797
**Betaine uptake**	
Betaine/carnitine/choline transporter (*bcct*)	OM942798, OM942799

## Data Availability

The authors confirm that all data underlying the findings are fully available without restriction. All the gene accession numbers mentioned in the text are available through the National Center for Biotechnology Information databases and listed in Table S1.

## Supplementary Materials

The following supporting information can be downloaded at: https://www.mdpi.com/article/10.3390/genes13060939/s1, Table S1: Primer sequences of osmoadaptive genes.
