# Supplementary material for: The Osmoprotectant Switch of Potassium to Compatible Solutes in an Extremely Halophilic Archaea Halorubrum kocurii 2020YC7
Source: Genes (Basel). 2022 May 24;13(6):939. doi: 10.3390/genes13060939 (PMC9222508; doi:10.3390/genes13060939)
Supplement: Supplementary file 1 [file genes-13-00939-s001.zip › genes-1658012-supplementary.pdf]

Table S1 Primer sequences of osmoadaptive genes

**Gene Name**	**Primer Sequences**
TrkH	F: CGGCTTTATCCTCTTTGAC<br>R: CGAAGTCCCTGATAAGCGA
TrkA	F: CCCCGCGTTGTAGAGAAAG<br>R: TCGAAGACGGTGACGTAAT
Kch	F: TGGGTGACCGTCAGAGCAT<br>R: GGTTAGCGGGAGCAGTATC
KefB	F: CGACGGAGTACAACCACGAGATC<br>R: GAGCCCGATCCAGAAGAAGAACAC
TreS	F: FGCTGTTCAAAGAGATGCGG<br>R: CCGCAGGAAGTTCACCCAG
BCCT family transport gene	F: TTCCTGCTCTTGCTGGGTT<br>R: ATGCGGGCAATGAACAGTC
SugA	F: TCTACGAAGAGGCGGATGC<br>R: GGGAAGCGTGTCGTAGATA
16s	F: CAGCCCACAATCCGAACTA<br>R: GAGACCCGCATCCTTACTT
